# High-level expression and molecular characterization of a recombinant prolidase from *Escherichia coli *NovaBlue

**DOI:** 10.7717/peerj.5863

**Published:** 2018-10-31

**Authors:** Tzu-Fan Wang, Meng-Chun Chi, Kuan-Ling Lai, Min-Guan Lin, Yi-Yu Chen, Huei-Fen Lo, Long-Liu Lin

**Affiliations:** 1 Department of Applied Chemistry, National Chiayi University, Chiayi, Taiwan; 2 Department of Food Science and Technology, Hungkuang University, Taichung, Taiwan; 3 Institute of Molecular Biology, Academia Sinica, Taipei, Taiwan

**Keywords:** *Escherichia coli*, Gene expression, Prolidase, Organic co-solvents, Chaotropic agent-induced denaturation, Molecular characterization

## Abstract

Long-term use of organophosphorus (OP) compounds has become an increasing global problem and a major threat to sustainability and human health. Prolidase is a proline-specific metallopeptidase that can offer an efficient option for the degradation of OP compounds. In this study, a full-length gene from *Escherichia coli* NovaBlue encoding a prolidase (*Ec*PepQ) was amplified and cloned into the commercially-available vector pQE-30 to yield pQE-*Ec*PepQ. The overexpressed enzyme was purified from the cell-free extract of isopropyl thio-β-D-galactoside IPTG-induced *E. coli* M15 (pQE-*Ec*PepQ) cells by nickel-chelate chromatography. The molecular mass of *Ec*PepQ was determined to be about 57 kDa by 12% sodium dodecyl sulfate–polyacrylamide gel electrophoresis and the result of size-exclusion chromatography demonstrated that the enzyme was mainly present in 25 mM Tris–HCl buffer (pH 8.0) as a dimeric form. The optimal conditions for *Ec*PepQ activity were 60 °C, pH 8.0, and 0.1 mM Mn^2+^ ion. Kinetic analysis with Ala-Pro as the substrate showed that the *K*_m _and *k*_cat_ values of *Ec*PepQ were 8.8 mM and 926.5 ± 2.0 s^−1^, respectively. The thermal unfolding of *Ec*PepQ followed a two-state process with one well-defined unfolding transition of 64.2 °C. Analysis of guanidine hydrochloride (GdnHCl)-induced denaturation by tryptophan emission fluorescence spectroscopy revealed that the enzyme had a [GdnHCl]_0.5,N-U _value of 1.98 M. The purified enzyme also exhibited some degree of tolerance to various water/organic co-solvents. Isopropanol and tetrahydrofuran were very detrimental to the enzymatic activity of *Ec*PepQ; however, other more hydrophilic co-solvents, such as formamide, methanol, and ethylene glycol, were better tolerated. Eventually, the non-negative influence of some co-solvents on both catalytic activity and structural stability of *Ec*PepQ allows to adjust the reaction conditions more suitable for *Ec*PepQ-catalyzed bioprocess.

## Introduction

Prolidases (Xaa-Pro dipeptidases, EC 3.4.13.9) act to hydrolyze all dipeptides with a non-polar amino acid at the N-terminus and proline or hydroxyproline at the C-terminus (Lowther & Matthews, 2002). This type of enzyme is widespread in nature and has been isolated from different mammalian tissues (Sjöström, Noŕen & Josefsson, 1973; Browne & O’Cuinn, 1983; Endo et al., 1987; Lupi et al., 2006) as well as from a variety of microorganisms, including the species of *Escherichia* (Park et al., 2004), *Xanthomonas* (Suga et al., 1995), *Lactobacillus* (Fernandez-Espla, Martin-Hernandez & Fox, 1997; Huang & Tanaka, 2015), *Pyrococcus* (Ghosh et al., 1998; Theriot et al., 2010), and *Aspergillus* (Jalving et al., 2002). Although the exact physiological role of prokaryotic prolidases remains to be elucidated, eukaryotic enzymes are thought to be involved in the recycling of collagen-derived proline (Hu, Phang & Valle, 2008). In this regard, activity fluctuations in eukaryotic-type prolidase can serve as an indicator of dysfunctional collagen metabolism as well as disease progression (Kitchener & Grunden, 2012; Namiduru, 2016).

As one of the most important components in pesticides and chemical warfare agents, organophosphorus (OP) compounds are highly toxic toward mammalian species (Kumar et al., 2010) and usually difficult to degrade through the natural metabolic processes of microorganisms (Karpouzas & Singh, 2006). Two well-known examples of OP compounds are the acetylcholinesterase inhibitor, diisopropylfluorophosphate, and G-series nerve agents, such as sarin and soman (Ganesan, Raza & Vijayaraghavan, 2010). Several microbial enzymes, particularly organophosphorus acid anhydrolase (OPAA) and phosphotriesterase, have been biochemically characterized for their high activity and unique substrate specificity against G-series nerve agents (Singh, 2009; Kitchener & Grunden, 2012). A recent review has documented that the three-dimensional structures of organophosphate-degrading metallohydrolases have a common “pita-bread” fold with a dinuclear metal center coordinated by five highly conserved residues (Schenk et al., 2016). Interestingly, the crystal structures of microbial prolidases also share the same “pita-bread” fold with the characteristic catalytic dinuclear metal site (Maher et al., 2004; Jeyakanthan et al., 2009; Weaver et al., 2014; Kgosisejo et al., 2017). Examination of the crystal structure of the nerve agent degrading OPAA/prolidase from *Alteromonas* sp. strain JD6.5 further helps us to understand the structural conservation between OPAA and prolidase (Vyas et al., 2010). Over the last two decades, some important advances in our understanding of the biotechnological application of prolidases have already been exerted on their intrinsic ability to hydrolyze toxic OP-containing compounds (DeFrank & Cheng, 1991; Cheng et al., 1998; DiTargiani et al., 2010; Theriot et al., 2011; Chandrasekaran, Belinskaya & Saxena, 2013; Yuh et al., 2017). This fact renders prolidases very suitable to act as a catalytic bioscavenger for alleviating OP nerve agent toxicity (Theriot et al., 2010; DiTargiani et al., 2010; Theriot et al., 2011).

Previously, some catalytic properties of *Escherichia coli* BL21 (DE3) prolidase (*Ec*PepQ) has been characterized (Park et al., 2004). The recombinant enzyme exhibits hydrolytic activity against a series of OP triesters so that it has been proposed to be useful for the kinetic resolution of racemic phosphate esters. Structural basis for the substrate selectivity of *Ec*PepQ has also been elucidated just a few years ago (Weaver et al., 2014). Through docking simulations and site-directed mutagenesis, Weaver and his co-workers have suggested that the location of the loop R370 in *Ec*PepQ plays an important part in the evolution of enzyme selectivity. Until now, to the best of our knowledge, only the aforementioned studies have investigated the molecular properties of *E. coli* prolidase. Given the fact that more information about the enzymatic characteristics of *Ec*PepQ is essential to ensure a satisfactory application of this enzyme, a full-length gene encoding *E. coli* NovaBlue prolidase was amplified and cloned into the pQE-30 expression vector. The overexpression of *Ec*PepQ was induced with isopropyl thio-β-D-galactoside (IPTG) and the recombinant enzyme was purified from the cell-free extract by affinity chromatography on nickel-nitrilotriacetate (Ni-NTA) resin. In addition, the biochemical and biophysical properties of *Ec*PepQ were successively examined in order to explore its biotechnological potential.

## Materials and Methods

### Expression and purification of the recombinant enzyme

Chromosomal DNA from *E. coli* NovaBlue was isolated by a commercial kit (Geno *Plus* Genomic DNA Extraction Miniprep System; Viogene Inc., Valencia, CA, USA) and used as a template for the amplification of the *pepQ* gene by polymerase chain reaction (PCR). PCR amplification was performed on Applied Biosystems 2400 Thermal Cycler (Thermo Fisher Scientific Inc., Waltham, MA, USA) with two sets of primers (PepQ-F: 5′-GGATCCATGGAATCACTGGCCTCGCTC and PepQ-R: 5′-AAGCTTTCACGCCAGTTTCAGATCCCG), designed on the basis of the gene sequence (Accession number: P21165) deposited in GenBank. Touchdown PCR was conducted as follows: an initial denaturation at 94 °C for 3 min, followed by 30 cycles of denaturation of 94 °C for 2 min, annealing at 53 °C for 1.5 min, and extension at 72 °C for 2 min. After a final extension at 72 °C for 2 min, the amplified fragment was digested with *Bam*HI and *Hin*dIII, and inserted into the predigested vector pQE-30 (Qiagen, Hilden, Germany) to yield pQE-*Ec*PepQ.

Single colony of *E. coli* M15 (pQE-*Ec*PepQ) was inoculated to Luria-Bertani (LB) medium supplemented with 100 μg/mL ampicillin and 25 μg/mL kanamycin and the culture was shaken at 37 °C and 180 rpm until the optical density at 600 nm reached 0.6. High-level expression of *Ec*PepQ in *E.coli* cells using a T5 promoter-based expression system in 100 mL of antibiotic-containing *LB* medium was initiated by adding IPTG to a final concentration of 100 μM. After incubation at 25 °C for 12 h, the culture cells were harvested by centrifugation, and were resuspended in 12.5 mL of buffer A (50 mM NaH_2_PO_4_, 300 mM NaCl, and 10 mM imidazole) and disrupted by sonication. The cell-free extract was subsequently applied to Ni-NTA agarose (Qiagen, Hilden, Germany) in a column, which had been equilibrated by buffer A. Afterward, the column was washed once with 30 mL of buffer A and eluted with buffer B (50 mM NaH_2_PO_4_, 300 mM NaCl, and 250 mM imidazole). Fractions enriched in protein and prolidase activity were pooled for gel electrophoresis and activity assay. Protein concentration was determined using the Bio-Rad protein assay kit with bovine serum albumin as the standard.

### Gel electrophoresis and size-exclusion chromatography

Sodium dodecyl sulfate–polyacrylamide gel electrophoresis (SDS–PAGE) was performed on a 12% polyacrylamide separating gel and a 5% stacking gel. For native gel electrophoresis, the experiment was carried out in a vertical slab gel apparatus with 5% stacking gel and 10% separating gel concentration. Coomassie blue R-250 dye was used to visualize protein bands on the polyacrylamide gels.

The apparent molecular mass of the native form of *Ec*PepQ was determined by gel filtration on a Superdex 200 HiLoad 16/60 column (GE Healthcare, Chicago, IL, USA) with 25 mM Tris–HCl buffer (pH 8.0) containing 10 mM Mg(OAc)_2_ and two mM DTT. A calibration curve was simultaneously established with four reference proteins, including thyroglobulin (670 kDa), aldolase (158 kDa), ovalbumin (43 kDa), and ribonuclease A (13.7 kDa). The elution volumes of reference proteins and the native enzyme were individually recorded to calculate their respective *K*_av_ values through the following equation:

$${K_{{\rm{av}}}} = {\rm{ }}({V_{\rm{e}}}-{V_{\rm{o}}}){\rm{ }}/{\rm{ }}({V_{\rm{t}}}-{V_{\rm{o}}})$$

Where *K*_av_, *V*_e_, *V*_o_, and *V*_t_ donate the distribution coefficient of a particular protein, the elution volume of a particular protein, the column void volume, and the total bed volume, respectively.

### Activity assays

Prolidase activity was measured by determining the release of proline from Ala-Pro by the recombinant enzyme. The amount of proline in the reaction mixture was estimated by the colorimetric method reported elsewhere (Ghosh et al., 1998). At the beginning of the enzyme reaction, *Ec*PepQ together with 25 mM Tris–HCl buffer (pH 8.0) and other reaction components (one mM Ala-Pro and 0.1 mM Mn^2+^) were warmed up separately at 60 °C for a short period of time (∼2 min). The catalytic action was initiated by adding 0.1 mL of a suitable dilution of enzyme to the reaction components and sufficient distilled water to bring the final volume up to 0.5 mL. The reaction mixture was incubated at 60 °C for 10 min and then stopped the catalytic action by adding 0.5 mL of acetic acid (3.5 N) and 0.5 mL of ninhydrin reagent (3%, w/v; 3.0 g ninhydrin in a mixture of 60 mL of glacial acid and 40 mL of phosphoric acid). Afterward, the resultant solution was heated at 100 °C for 10 min. The amount of substrate hydrolyzed was calculated from the increase in absorbance at 515 nm. One unit of *Ec*PepQ activity is defined as the amount of enzymes that catalyze the release of one μmole of proline from Ala-Pro per minute at 60 °C and pH 8.0.

Effect of different temperatures (4–80 °C) on the prolidase activity was evaluated with 25 mM Tris–HCl buffer (pH 8.0). The thermal stability of *Ec*PepQ was studied at pH 8.0 and in the temperature range of 4–80 °C for 10 min, and the residual activity was immediately measured under the standard assay conditions. The pH optimum of *Ec*PepQ was determined at 60 °C using 25 mM phosphate-citrate buffer (pH 2.8–5.0), 25 mM sodium acetate buffer (pH 3.6–5.9), 25 mM sodium citrate buffer (pH 4.0–6.0), 25 mM sodium dihydrogen phosphate buffer (pH 6.0–8.0), 25 mM Hepes-NaOH buffer (pH 6.8–8.2), 25 mM Tris–HCl buffer (pH 7.0–9.0), and 25 mM glycine-NaOH buffer (pH 8.6–10.6). The pH stability of *Ec*PepQ was evaluated at 30 °C for 30 min by incubating the enzyme sample (10 μg/mL) at different buffer systems, and the residual activity was then measured under the standard assay conditions. Stimulation of *Ec*PepQ by metal cofactor was determined through assaying the enzymatic activity in presence of 1.0 mM metal ions.

Kinetic parameters of *Ec*PepQ for the hydrolysis of Ala-Pro were determined using 45 μg/mL of the purified enzyme. Substrate concentrations ranging from 0.2 to 14.2 mM were applied to the reaction mixture. The assays were performed at 60 °C for 10 min in Eppendorf tubes in a total volume of 0.5 mL. To determine the kinetic constants of *Ec*PepQ, a Lineweaver–Burk plot was created with data points derived from double-reciprocal transformation.

### Circular dichroism spectroscopy

The circular dichroism (CD) spectra of enzyme samples were recorded in the far-UV region (190–260 nm) on the JASCO J-815 spectropolarimeter (JASCO Corporation, Tokyo, Japan) with the light path of one mm. Prior to spectral analysis, the desalted enzyme sample at a final concentration of 0.2 mg/mL was individually incubated at the indicated conditions or treated with different types of organic co-solvents (5–70%, v/v) for 30 min. The spectra were recorded at room temperature (except for the experiment of heat-induced denaturation) using an external circulating water bath. Each spectrum was an average of at least 10 scans and the control signals were taken and subtracted from the respective test to minimize the chances of any false signal generation due to the salts, chaotropic agents or solvents. The data were expressed as mean residue molar ellipticity (deg·cm^2^·/dmol) based on a residue number of 443 and a mean residue weight (MRW) of 50,176 Da. Mean residue molar ellipticity can be calculated as follows: [θ]_mrw_ = MRW × θ_obs_/10 × *c* × *l*, where θ_obs_ is the observed ellipticity in degree at a given wavelength, *c* is the protein concentration in mg/mL, and *l* is the pathlength in cm. The spectra were also quantitatively analyzed by the DICHROWEB server to estimate the content of secondary structure elements (Lobley, Whitmore & Wallace, 2002).

Thermal unfolding of *Ec*PepQ samples (∼0.15 mg/mL) in 25 mM Tris–HCl buffer (pH 8.0) was determined by monitoring the temperature-dependent changes of the molar ellipticity at 222 nm. In this experiment, *Ec*PepQ samples were heated at scan rates of 0.5, 1, and 2 °C/min. The alterations in molar ellipticity at 222 nm were further fitted with a two-state model (Pace, 1990) to acquire the midpoint (*T*_m_) of the unfolding curve. To explore the possible refolding of denatured *Ec*PepQ, the temperature of thermoelectrically controlled cell holder was reduced by 0.5 °C/min and measurements were taken once every minute.

### Fluorescence spectroscopy

Fluorescence analysis of *Ec*PepQ samples was performed at 25 °C in a JASCO FP-6500 fluorescence spectrophotometer with an excitation wavelength of 295 nm. All fluorescence spectra were corrected for the contribution of 25 mM Tris–HCl buffer (pH 8.0), chaotropic agent and organic co-solvents. Before fluorescence spectroscopy, the purified *Ec*PepQ was individually mixed with an appropriate amount of buffer and various amounts of guanidine hydrochloride (GdnHCl) or organic co-solvents to produce solutions with a protein concentration of approximately 0.15 mg/mL. Fluorescence spectra were then recorded for these samples after 30 min of equilibration at room temperature. The emission profiles of *Ec*PepQ samples were recorded from 305 to 500 nm at a scanning rate of 240 nm/min. The maximal peak of fluorescence spectra and the changes in fluorescence intensity were brought together to calculate the average emission wavelength (AEW) (λ) according to Eq. (1) (Royer, Mann & Matthews, 1995).

(1) $$\left\langle \lambda  \right\rangle  = {{\sum\nolimits_{i = {\lambda _1}}^{{\lambda _N}} {\left( {{F_i} \cdot {\lambda _i}} \right)} } \over {\sum\nolimits_{i = {\lambda _1}}^{{\lambda _N}} {\left( {{F_i}} \right)} }}$$

in which *F*_i_ is the fluorescence intensity at the specific emission wavelength (λ_i_).

To calculate the transition point and Δ*G*_N-U_ of GdnHCl-treated *Ec*PepQ_,_ the unfolding data were further fitted with Eq. (2) (Pace, 1990).

(2) $${y_{obs}} = {{{y_N} + {y_U}\ \bullet \  {e^{ - \left( {{{\Delta {G_{{{({H_2}O)}_{N \to U}}}} - {m_{N \to U}}[GdnHCl]} \over {RT}}} \right)}}} \over {1 + {e^{ - \left( {{{\Delta {G_{({H_2}O)N \to U}} - {m_{N \to U}}[GdnHCl]} \over {RT}}} \right)}}}}$$

where *y*_obs_ represents the observed biophysical signal, *y*_N_ and *y*_U_ are the calculated signals of the native and unfolded states, respectively, [*GdnHCl*] is the concentrations of the chaotropic agent, Δ*G*_N-U_ is the free energy change for the *N*↔*U* process, and the *m*_N-U_ represents the sensitivity to denaturant concentration.

## Results

### Enzyme expression and purification

**Figure 1:**
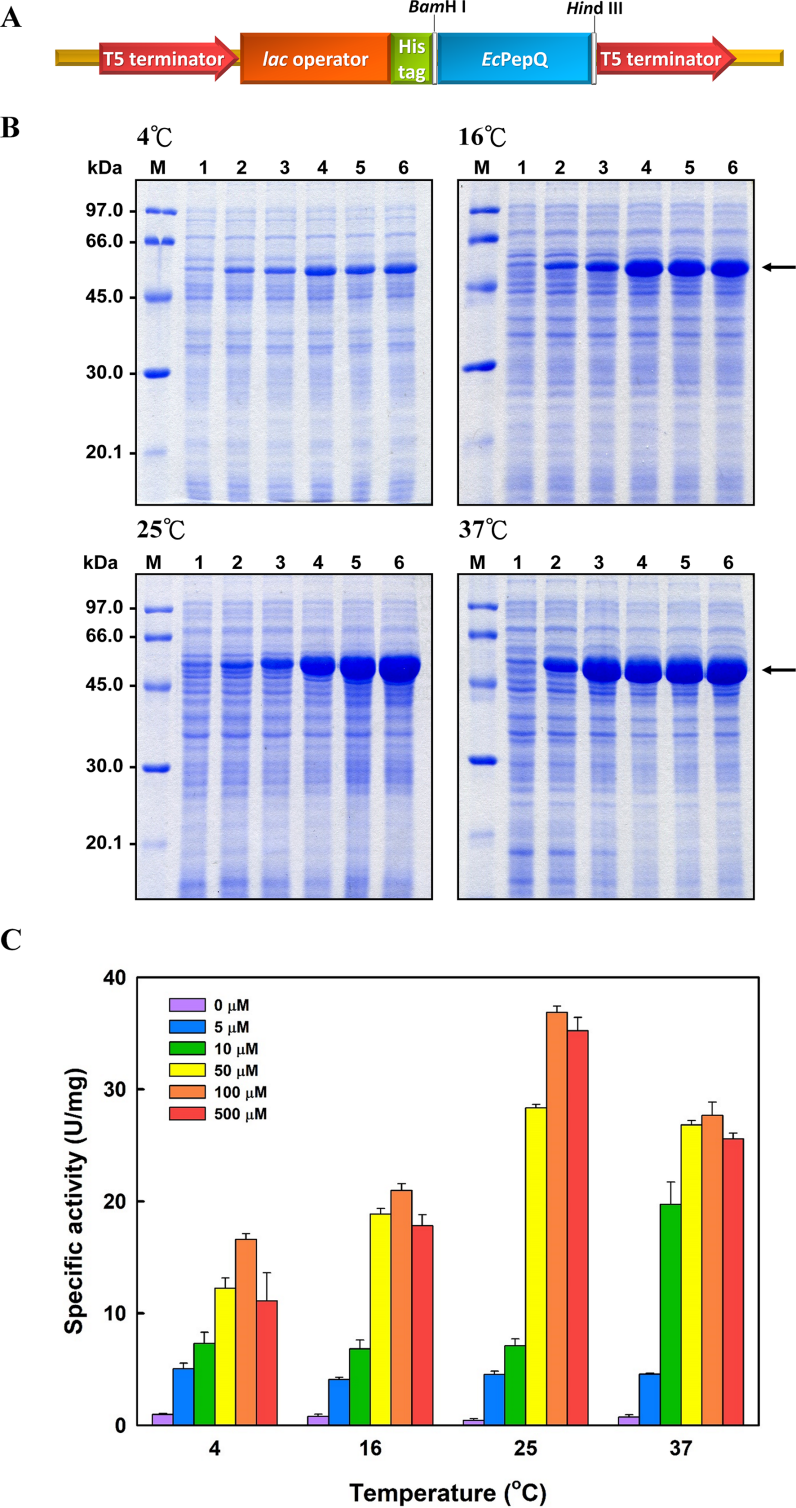
Analyses of the soluble proteins and specific activity of *E. coli* M15 (pQE-*Ec*PepQ) under a specific condition. (A) Schematic diagram of the key elements of pQE-*Ec*PepQ. (B) Analysis of the crude extracts by SDS–PAGE. Lanes: M, protein size markers; 1, cell growth without IPTG induction; 2, cell growth with 5 μM IPTG induction; 3, cell growth with 10 μM IPTG induction; 4, cell growth with 50 μM IPTG induction; 5, cell growth with 100 μM IPTG induction; 6, cell growth with 500 μM IPTG induction. (C) Effects of incubation temperature and IPTG concentration on the production of active *Ec*PepQ. The amount of active enzyme was determined by measuring the prolidase activity of the soluble extracts shown in (B). These data were a representative of three independent experiments.

In order to overproduce *Ec*PepQ, the PCR-amplified DNA fragment encoding an approximately 57 kDa protein was digested with *Bam*HI and *Hin*dIII, and cloned into the expression vector pQE-30 to yield pQE-*Ec*PepQ (Fig. 1A). Such construction allows the expressed *Ec*PepQ bearing 10 additional amino acid residues at its N-terminus, which facilitates the single-step purification of the recombinant protein by metal-affinity chromatography. Initially, enzyme production by *E. coli* M15 (pQE-*Ec*PepQ) was evaluated at 28 °C in a five mL medium containing essential antibiotics and 100 μM IPTG for a period of 24 h to select an appropriate time for further optimization. With this information, we then investigated effects of temperature and inducer concentrations on the production of active enzyme by *E. coli* M15 (pQE-*Ec*PepQ). In these experiments, one mL of bacterial culture was harvested and disrupted by sonication, and the cell-free extract was subsequently analyzed for its specific activity toward Ala-Pro. Results demonstrated that the production of functional *Ec*PepQ achieved a maximum upon a 12 h induction of *E. coli* M15 (pQE-*Ec*PepQ) with 100 μM IPTG. One major band corresponding to a molecular mass of about 57 kDa was clearly observed in the cell-free extracts of IPTG-induced recombinant cells when the cultivation temperatures were set at above 16 °C (Fig. 1B). It is noteworthy that less amount of the recombinant enzyme were produced by *E. coli* M15 (pQE-*Ec*PepQ) cultivated at 4 °C. Apparently, the optimal IPTG concentration for the production of active *Ec*PepQ was 100 μM (Fig. 1C). Based on these observations, the best conditions for the high-level production of active *Ec*PepQ by the recombinant cells were the cultivation of *E. coli* M15 (pQE-*Ec*PepQ) cells at 25 °C, an inducer concentration of 100 μM, and a growth period of 12 h. Under the aforementioned conditions, the specific activity of the cell-free extract from *E. coli* M15 (pQE-*Ec*PepQ) reached 36.9 U/mg.

**Figure 2:**
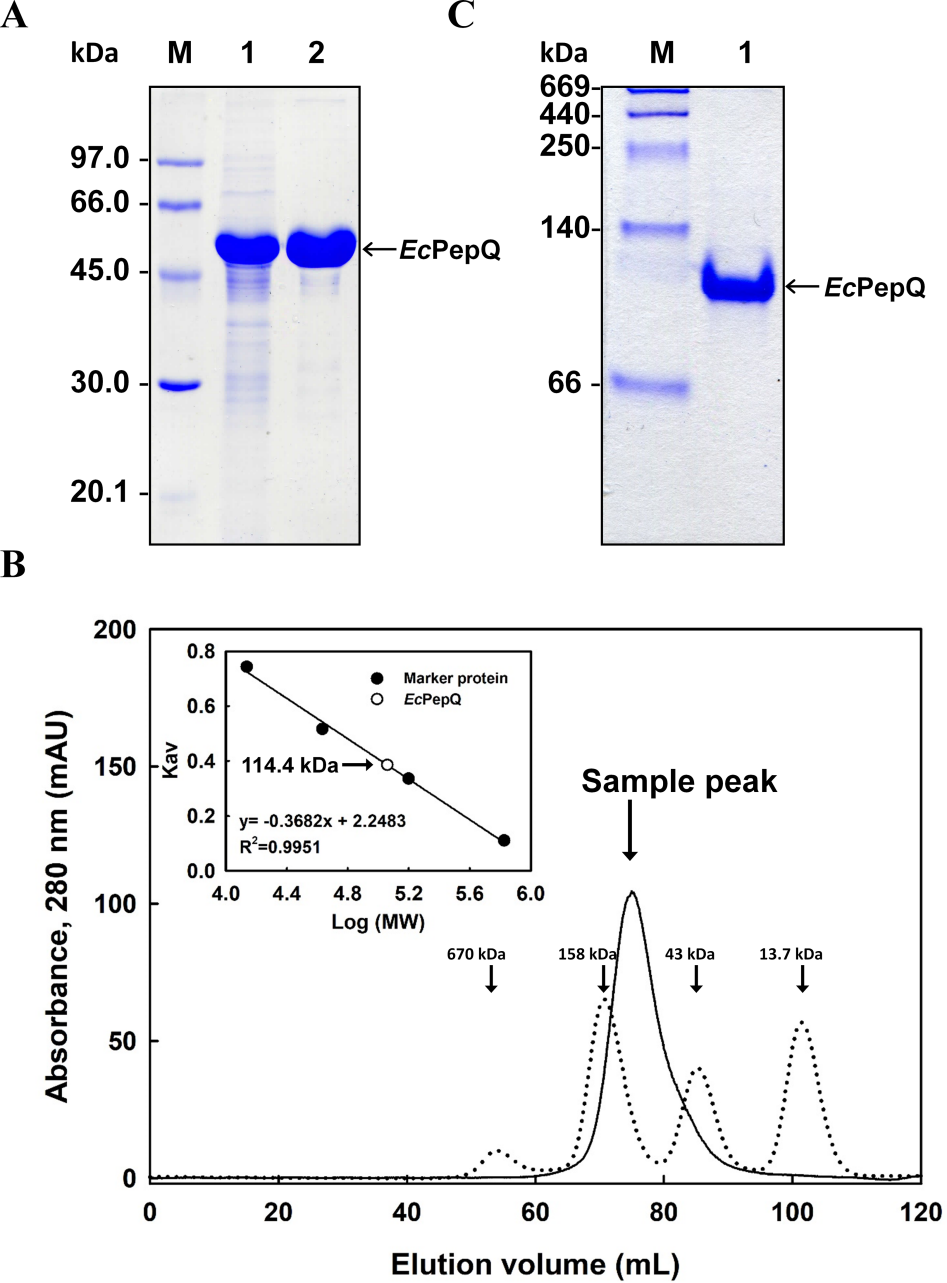
Gel electrophoresis and size-exclusion chromatography of the recombinant enzyme. (A) SDS–PAGE analysis. Lanes: M, protein size markers; 1, the crude extract of *E. coli *M15 (pQE-*Ec*PepQ); 2, the enzyme sample after Ni-NTA purification. (B) FPLC analysis. Blue dextran 2000 was used to determine the void volume. The *K*_av_ values for the standard proteins and *Ec*PepQ were plotted against the logarithm of their molecular weights to estimate the native molecular mass of *Ec*PepQ. (C) Native PAGE analysis. Lanes: M, protein size markers; 1, the enzyme sample after Ni-NTA purification.

The recombinant enzyme in the cell-free extract of *E. coli* M15 (pQE-*Ec*PepQ) cells was further purified to near homogeneity by an affinity procedure that uses Ni-NTA resin. SDS–PAGE analysis of the pooled fractions exhibited a predominant band with a molecular mass of approximately 57 kDa (Fig. 2A), a finding which is in close agreement with the value deduced from the gene sequence of *Ec*PepQ. The His_6_-tagged enzyme could be purified nearly16-fold with a yield of 68.4% by single-step affinity chromatography. The native state of *Ec*PepQ was also determined by fast performance liquid chromatography (FPLC) gel filtration (Fig. 2B). The experimental result showed that *Ec*PepQ was eluted from the HiLoad 16/600 Superdex@200 PG column just after aldolase (158 kDa), in a fraction corresponding to a calculated molecular mass of 114.4 kDa (Fig. 2B). Owing to gel electrophoresis analysis of the purified enzyme suggested only one type of subunit (Figs. 2A and 2C), the native state of *Ec*PepQ is most likely to be composed of two identical 57 kDa subunits.

### Biochemical characterization of *Ec*PepQ

**Figure 3:**
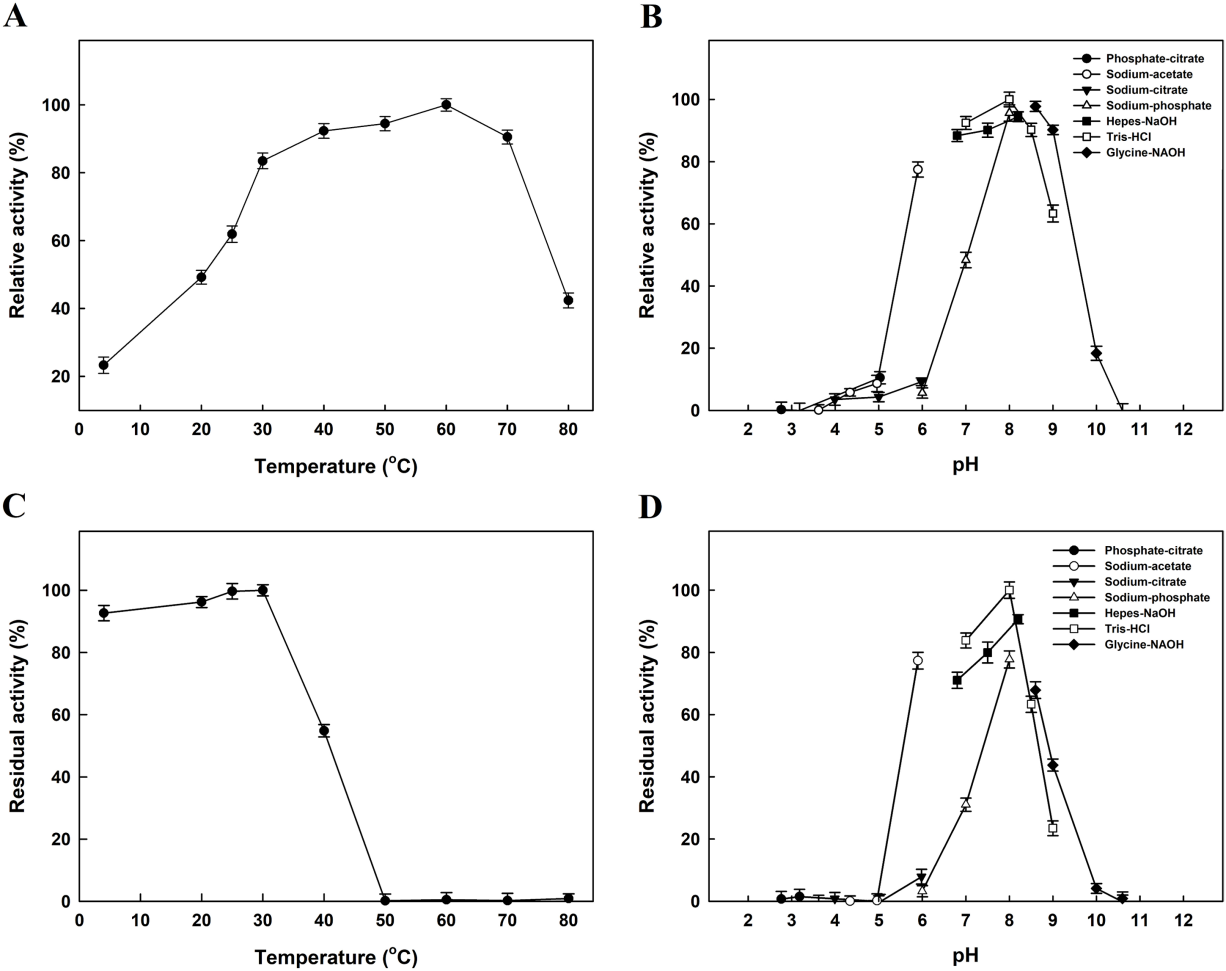
Effects of temperature and pH on activity (A and B) and stability (C and D) of *Ec*PepQ. Enzyme assay was performed as aforementioned procedures with one mM Ala-Pro as the substrate. 100% relative activity refers to the percentage of the initial reaction rate obtained by the enzyme at pH 8.0 and 60 °C. The residual activity was expressed as a percentage of specific activity with the untreated sample being defined as 100%. The data are expressed as mean ± SD of three independent experiments.

The effect of temperature on *Ec*PepQ activity was investigated at pH 8.0 over a temperature range from 4 to 80 °C. As shown in Fig. 3A, the maximum activity for *Ec*PepQ was at 60 °C. The pH effect was also studied at 60 °C in the range of 2.7–11.0 (Fig. 3B). The maximum activity was observed at pH 8.0, whereas the enzyme was sensitive to pH shift with more than 80% of activity attenuation at pH 5.0 and 10.0. The thermostability of *Ec*PepQ was evaluated by incubating the enzyme at temperatures between 4 °C and 80 °C for 10 min. As shown in Fig. 3C, the enzyme displayed remarkable stability with more than 90% of the initial activity preserved at temperatures below 30 °C. However, there was a considerable decrease in its activity at temperatures greater than 40 °C. In addition, the effect of pH on the stability of *Ec*PepQ was investigated by incubating the enzyme at 30 °C and different pH’s for 1 h. The experimental results showed that it was stable in the pH value of 8.0, with 65–80% of the full activity at a pH range of 7.0–9.0 (Fig. 3D). However, the enzyme was almost inactive at pH values beyond 5.0 and 10.0.

**Figure 4:**
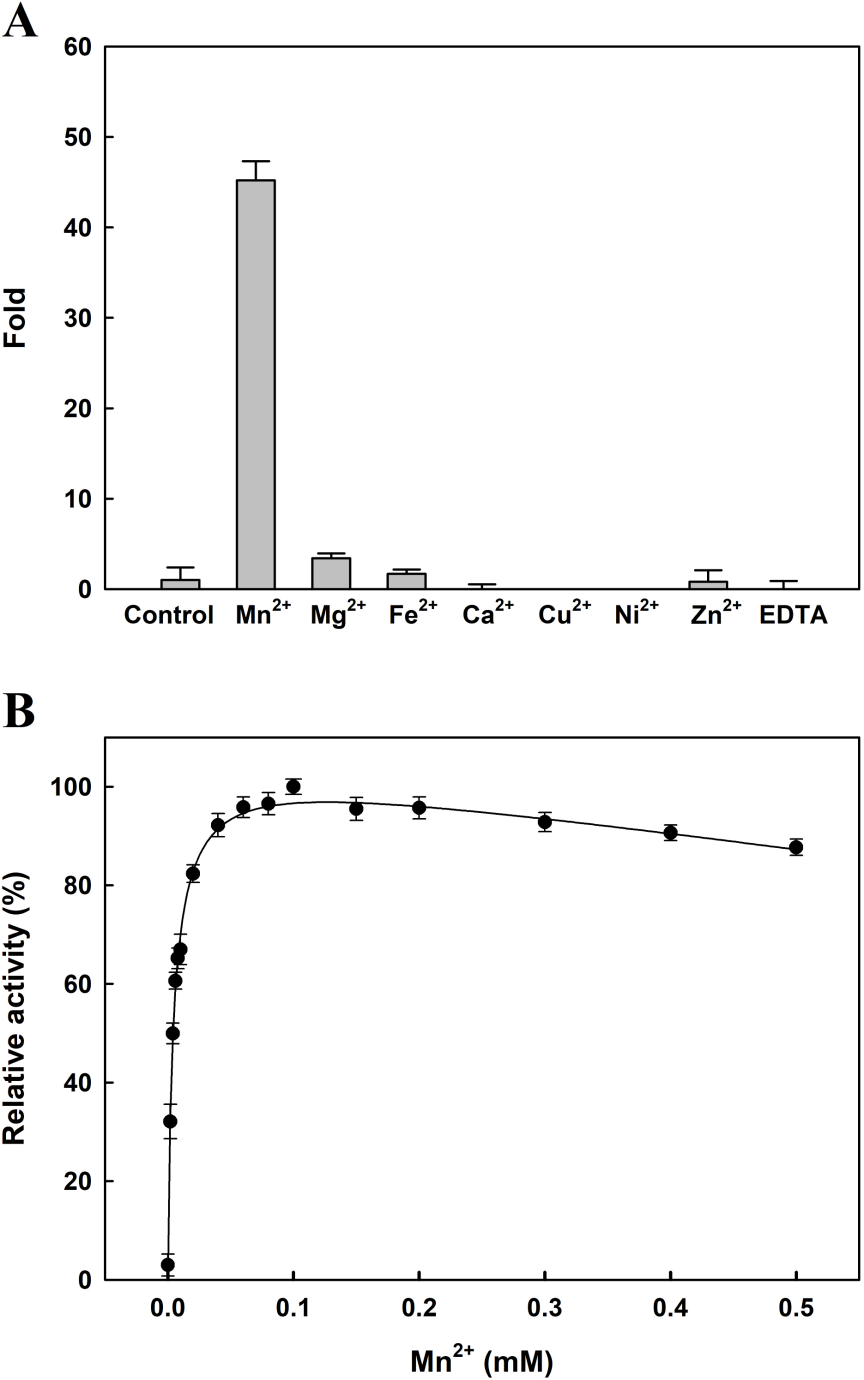
Effects of divalent metals (A) and different concentrations of Mn^2+^ ion (B) on the catalytic activity of *Ec*PepQ. In these experiments, the sample without extrinsic metal ions was used as a control and the EDTA-treated enzyme served as a negative reference. The data were expressed as mean ± SD of three independent experiments.

The effects of several metal ions on *Ec*PepQ activity at a final concentration of one mM are shown in Fig. 4A. It is noteworthy that the addition of Mn^2+^ ion into the reaction mixture greatly stimulated *Ec*PepQ activity, increasing it by approximately 43-fold as compared with the control. To verify the optimal concentration of Mn^2+^ ion, *Ec*PepQ activity was assayed under different concentrations of this metal ion. The results clearly indicated that Mn^2+^ ion at a final concentration of 0.1 mM had the greatest stimulation effect on the prolidase activity (Fig. 4B). However, the presence of Mg^2+^ and Fe^2+^ ions in the reaction mixture appeared to slightly enhance the enzymatic activity. Partial inhibition (∼1% inhibition) was observed in the presence of Zn^2+^ ion, and the strongest inhibitory effect was found in Cu^2+^ and Ni^2+^ ions.

The Michaelis–Menten kinetic constants, *K*_m_ and *V*_max_, for the purified *Ec*PepQ were determined by using varying concentrations of Ala-Pro. Prolidase activity was measured under standard assay conditions as described earlier and the obtained results were plotted as a graph of enzyme activity (U/mL) against concentration of substrate [μM], which yields a hyperbolic curve with *K*_m_ and *V*_max_ values. From the graph, *K*_m_ and *V*_max_ values of *Ec*PepQ were determined to be 8.8 ± 1.1 mM and 434.8 μM/min, respectively. A *k*_cat_ value of 926.5 ± 2.0 s^−1^ was further obtained through the Lineweaver–Burk plot.

### Spectroscopic studies

**Figure 5:**
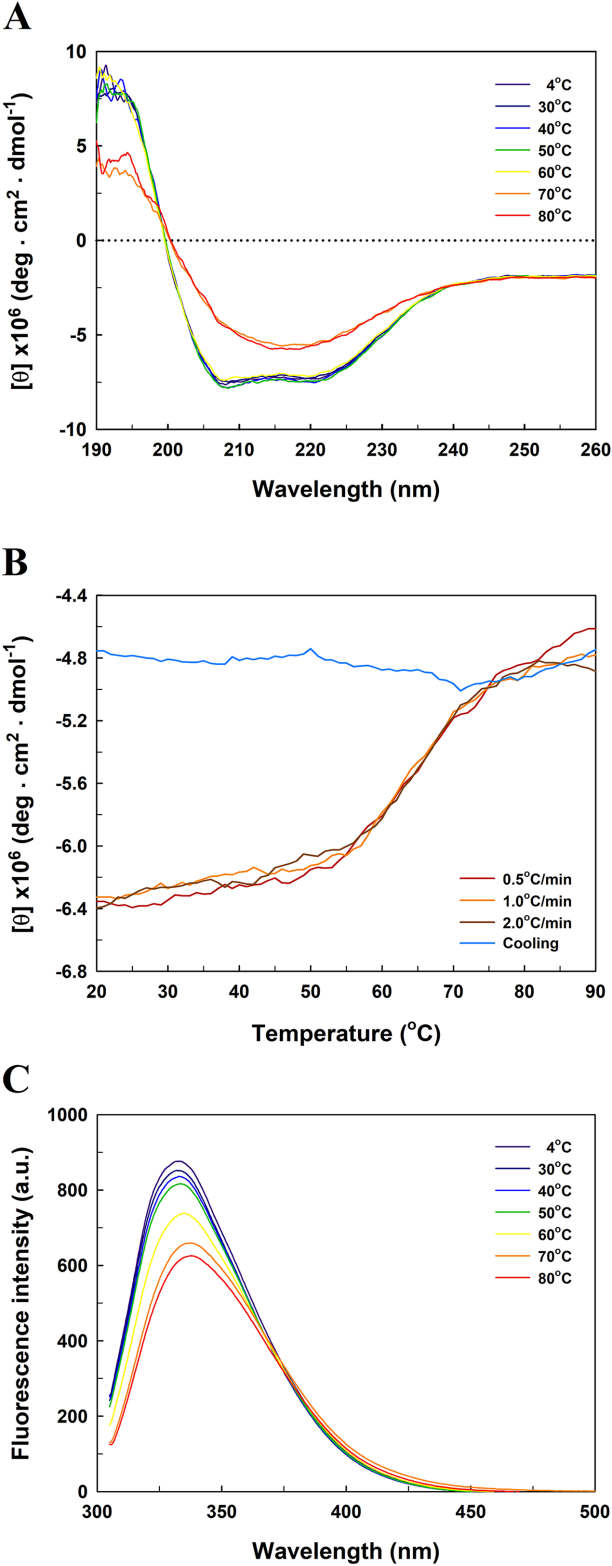
Far-UV CD and intrinsic tryptophan fluorescence spectra of *Ec*PepQ. (A) Temperature-dependent CD spectra of the enzyme. Far-UV CD spectra of the enzyme were recorded at the indicated temperatures over the wavelength range of 190–260 nm. (B) Transition and cooling curves of the enzyme . The temperature-induced unfolding of the enzyme was monitored at three different heating rates as aforementioned procedures. The blue line represents the cooling curve of the unfolded protein, which had been heated with a scan rate of 2.0 °C/min. (C) Temperature-dependent fluorescence spectra of the enzyme. Fluorescence spectra of the enzyme were recorded at the indicated temperatures over the emission wavelength range of 305–500 nm.

Figure 5 presents the far-UV CD spectra of *Ec*PepQ samples. The CD spectrum of the recombinant enzyme displays two strong peaks of negative ellipticity at 208 and 222 nm, indicative of a substantial α-helical content with lesser amounts of β-sheet and random coil (54% α-helix, 9% β-sheet and 21% random coil). The representative peaks for α-helix were significantly diminished at temperatures above 60 °C (Fig. 5A). The thermal unfolding of *Ec*PepQ was initiated at 50 °C and the CD signal at 222 nm was completely disappeared at the denaturation temperature of 75 °C (Fig. 5B). Also, it is evident that the thermal denaturation of *Ec*PepQ followed a two-state process with a well-defined *T*_m_ value of 64.2 ± 0.2 °C. To further explore whether the unfolding process was reversible or not, thermal denaturation of the enzyme sample was determined by monitoring the ellipticity at 222 nm at three constant heating rates. After the thermal denaturation went to completion, the enzyme samples were reversely cooled down to 20 °C using the same scan speed. Figure 5B shows the transition curves obtained with *Ec*PepQ solutions at heating rates of 0.5, 1, and 2 °C/min. It is clear that there were no significant differences between these heating rates with respect to the transition temperatures. Besides, it can be seen that the native secondary structure of the enzyme was not recovered immediately after the unfolded protein was cooled down from 90 to 20 °C (Fig. 5B). This observation indicates that the thermal denaturation of *Ec*PepQ is highly irreversible even at the very early stages of the unfolding process.

To probe the change in the tertiary structure of *Ec*PepQ as a function of temperature, the enzyme conformations were also analyzed by fluorescence spectroscopy. Figure 5C shows the intrinsic fluorescence spectra of *Ec*PepQ at different temperatures. Clearly, the fluorescence intensity of *Ec*PepQ was decreased by more than 16% when the enzyme samples were analyzed at temperatures above 60 °C. The tryptophan emission fluorescence spectrum for *Ec*PepQ was maximized at a wavelength of around 333.6 nm. It could be seen that the λ_max_ was individually displayed 1.2, 3.8, and 4.2 nm blue shift when the temperatures were set at 60, 70, and 80 °C (Fig. 5C). Together with the aforementioned CD data, it can be concluded that a significant change in the *Ec*PepQ structure has occurred as a consequence of temperature elevation.

### GdnHCl-induced denaturation of *Ec*PepQ

**Figure 6:**
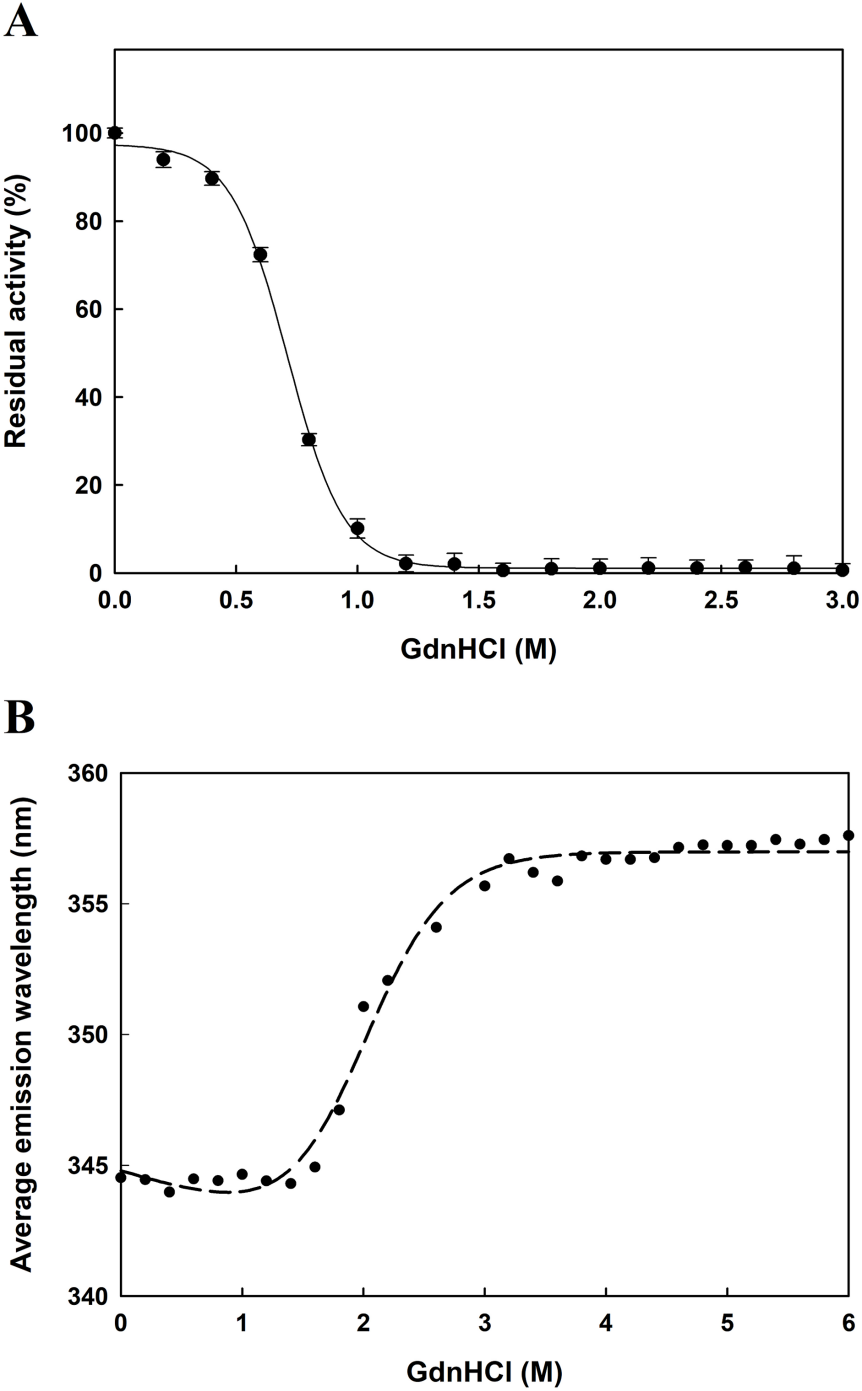
Concentration effect of GdnHCl on the catalytic activity of *Ec*PepQ (A) and the corresponding changes in the tertiary structure as monitored by AEW value (B). The purified enzyme at a final concentration of 0.15 mg/mL was incubated with different concentrations of GdnHCl at 30 °C for 10 min. Then, the sample solutions were subjected to measurement of prolidase activity under the standard assay conditions and fluorescence analysis.

The function of proteins depends on their ability to acquire a unique three-dimensional structure. It is generally known that GdnHCl acts as a classical denaturant to bring about unfolding of proteins by disrupting intramolecular interactions mediated by non-covalent forces. As shown in Fig. 6A, *Ec*PepQ treated with GdnHCl at concentrations of less than 0.5 M retained >80% of the prolidase activity. An increase in concentration up to 1.0 M resulted in 7% of the activity remaining, whereas the enzyme was completely inactivated after treatment with 1.2 M GdnHCl. These results suggest that GdnHCl concentrations below 0.5 M only cause a relatively small change in the molecular structure of *Ec*PepQ and its catalytic activity can tend to be recovered upon removal of the denaturant. Giving the fact that *Ec*PepQ contains a total of seventeen tryptophanyl residues, fluorescence spectroscopy is very suitable for its conformational study. Unfolding of this enzyme at different concentrations of GdnHCl was accordingly performed and the obtained data were shown in Fig. 6B. The AEW that reported on the changes in both fluorescence wavelength and fluorescence intensity was used to calculate the thermodynamic parameter of the unfolding process. As shown in Fig. 6B, *Ec*PepQ started to unfold at 1.2 M denaturant and exhibited a [GdnHCl]_0.5,N-U_ value of 1.98 M, which corresponds to a free energy change (Δ*G*_N-U_) of 6.18 kcal/mol.

### Effects of co-solvents on the catalytic activity and molecular structure of *Ec*PepQ

**Figure 7:**
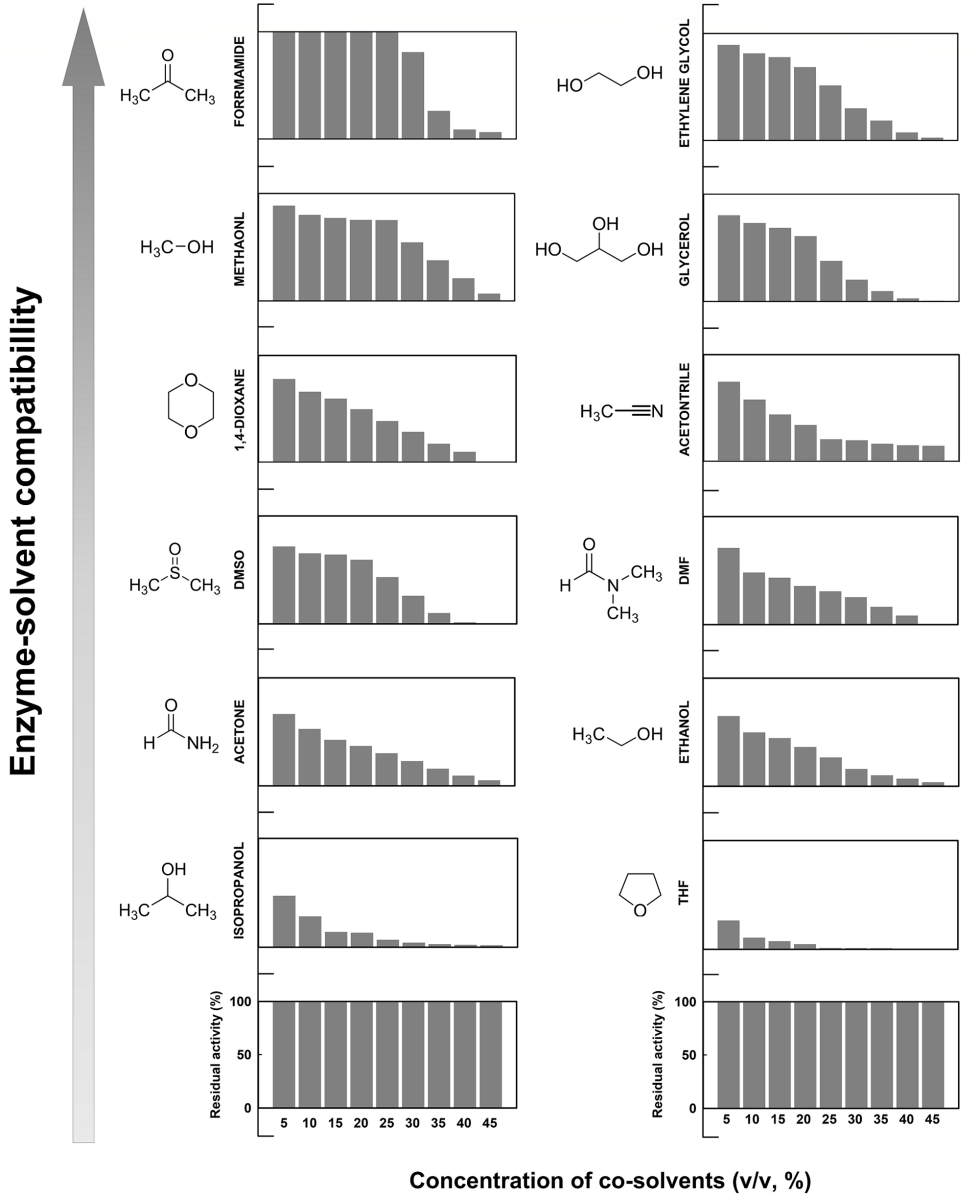
Effect of different water-miscible organic co-solvents on the enzymatic activity of *Ec*PepQ. The enzymatic activity was determined in the reaction mixture with different concentrations of organic co-solvents under the standard assay conditions. The residual activity was expressed as a percentage of specific activity with the solvent-free sample being defined as 100%.

Concentration effects of 12 different organic co-solvents on the enzymatic activity of *Ec*PepQ were investigated with the widely used substrate, Ala-Pro. As shown in Fig. 7, the enzyme exhibited distinct sensitivity to these organic co-solvents. It is noteworthy that the catalytic capability of *Ec*PepQ was well preserved in most of the organic co-solvents tested up to concentrations of 15% (v/v). Isopropanol and tetrahydrofuran (THF) clearly inactivated the enzyme at a concentration of 30% (v/v). Conversely, formamide, methanol, ethylene glycol, and glycerol were found to be the most compatible co-solvents to *Ec*PepQ (Fig. 7). Among the remaining co-solvents, the least level of disability to the functionality of *Ec*PepQ was dimethyl sulfoxide (DMSO), followed by 1,4-dioxane, acetonitrile, acetone and ethanol. It is also important to mention that the enzymatic activity of *Ec*PepQ was primarily decreased as a consequence of an increase in co-solvent concentrations (Fig. 7). Some literatures have shown that DMSO at low concentrations (<10%, v/v) actually promotes the stabilization of some proteins (Huang, Dong & Caughey, 1995; Arakawa, Kita & Timasheff, 2007; Batista et al., 2013). Although the protective mechanism of DMSO remains obscure, protein-solvent preferential interactions might be appropriate to interpret its beneficial effect on the conformational stability of proteins in aqueous solutions (Timasheff, 2002; Arakawa, Kita & Timasheff, 2007).

**Figure 8:**
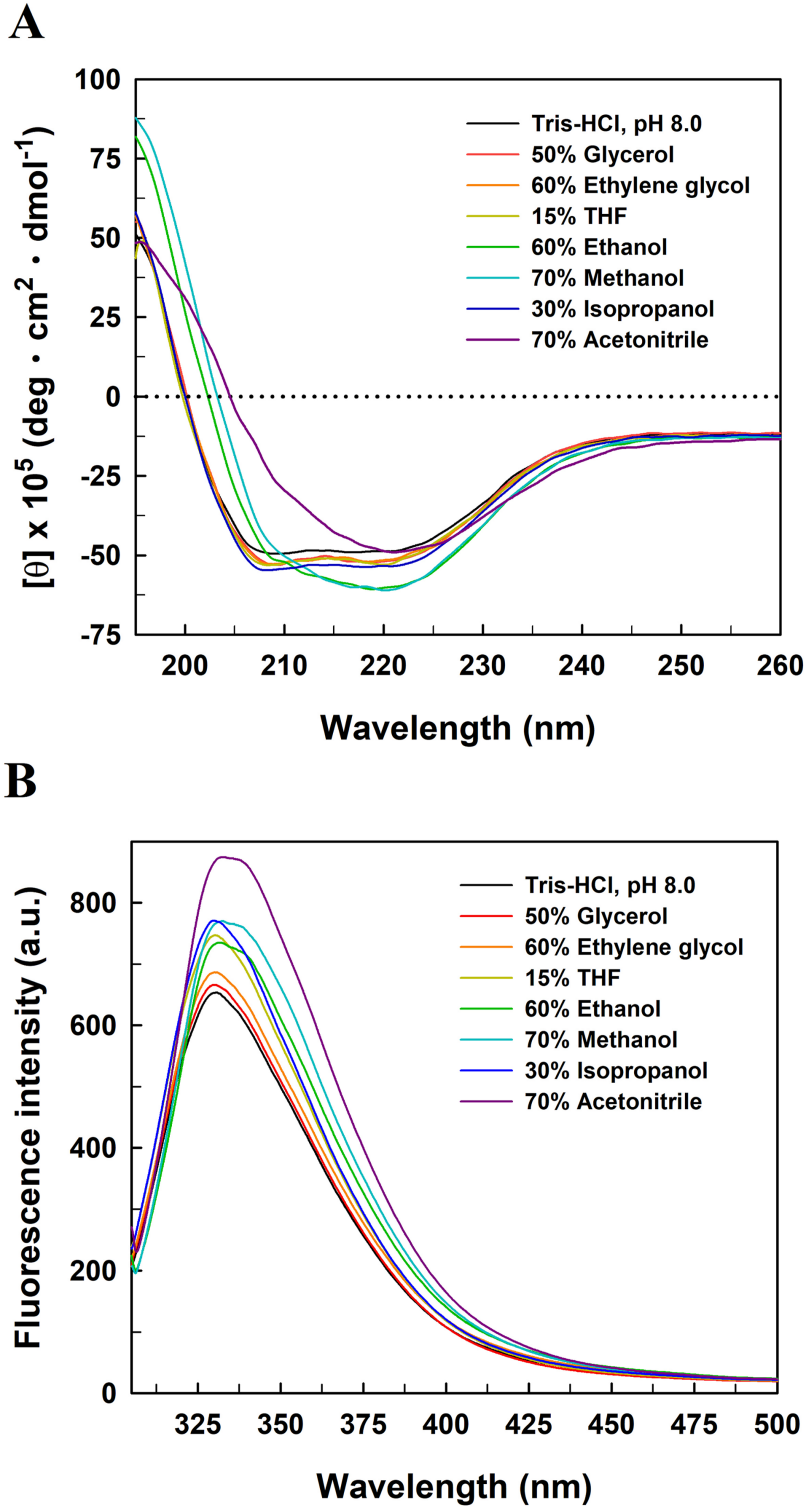
Far-UV CD (A) and intrinsic tryptophan fluorescence (B) spectra of *Ec*PepQ in the presence of water-miscible organic co-solvents. Spectral analyses of the enzyme were carried out at 25 °C in either 25 mM Tris–HCl buffer (pH 8.0) or the buffer supplemented with various organic co-solvents at concentrations that led to reductions in the catalytic activity of ≥90%.

To explore the relationship between the functional inactivation and structural disruption of *Ec*PepQ in the water/organic co-solvent mixtures, CD and fluorescence studies were carried out with our laboratory facilities. The far-UV CD spectra of *Ec*PepQ were essentially determined at the co-solvent concentrations that resulted in ≥90% decreases in the prolidase activity, and compared with that measured under solvent-free condition (*Ec*PepQ in 20 mM Tris–HCl buffer, pH 8.0). Considering the presence of DMSO, 1,4-dioxane, and formamide had strong negative interference on the ellipticity signals of *Ec*PepQ, these three co-solvents were excluded from the analysis of CD spectra. As the data presented above, the far-UV CD spectrum of the solvent-free enzyme sample displayed two strong peaks of negative ellipticity at 208 and 222 nm (Fig. 8A). The spectrometric characteristic of *Ec*PepQ was significantly diminished in the presence of water-miscible organic co-solvents, especially in the presence of 70% acetonitrile, 70% methanol, and 60% ethanol. These data clearly indicates that high concentrations of some organic co-solvents lead to profound alterations in the far-UV spectra, reflecting a substantial loss of the secondary structure of *Ec*PepQ.

Fluorescence emission spectra were also determined upon the excitation of the enzyme samples at 295 nm to monitor changes to the microenvironments of tyrosine and tryptophan residues (Royer, 2006). The fluorescence spectrum of solvent-free enzyme sample exhibited an emission maxima at 330.8 nm (Fig. 8B). Organic co-solvent concentrations that impaired the prolidase activity of *Ec*PepQ to less than 10% generally resulted in either a red shift or a blue shift in the maximum wavelength and a subtle increase in the emission intensity as well. Based on these facts, we speculate that at least some of the fluorescent residues of *Ec*PepQ undergo a relevant change in their local environments (Royer, 2006). Such results further enhance the idea that significant changes in the molecular structure of *Ec*PepQ have occurred upon treatment with high concentrations of organic co-solvents, in good agreement with the experimental results obtained from the analysis of CD spectra.

## Discussion

In this study, optimization of cultivation conditions for enzyme production by *E. coli* M15 (pQE-*Ec*PepQ) was achieved at an incubation time of 12 h, a final inducer concentration of 100 μM and an incubation temperature of 28 °C. Some previous reviews have already documented that the cultivation of recombinant *E. coli* cells at low temperature and the use of ideal inducer concentration might favor the production of functional proteins (Rosano & Ceccarelli, 2014; Kaur, Kumar & Kaur, 2018). Apparently, the ability to overexpress *Ec*PepQ by the recombinant cells and to purify the active enzyme in a large quantity allows for its molecular characterization and the development of a biochemical process for the remediation of OP compounds.

The structural and functional aspects of protein oligomerization have acquired growing importance over the last two decades. Oligomerization is usually essential for proteins to execute their biological functions and thus is a phenomenon crucial in triggering various physiological pathways (Hasimoto & Panchenko, 2010; Liu, 2015). The majority of protein oligomers forms through non-covalent weak associations, which can often lead to the assembly of subunits into metastable dimers or oligomers (Liu, 2015). A search of 452 human enzymes by Australian researchers has shown that most of these enzymes are present in oligomeric forms and only a third exists as monomers (Marianayagam, Sunde & Matthews, 2004). Based on the experimental results of gel electrophoresis and size-exclusion chromatography, it is apparent that *Ec*PepQ exists as a dimeric protein in aqueous solution. The dimeric status is consistent with earlier findings of various microbial prolidases (Ghosh et al., 1998; Jalving et al., 2002; Yang & Tanaka, 2008; Theriot et al., 2010; Weaver et al., 2014; Huang & Tanaka, 2015).

The reported prolidases exhibit metal-dependent activity, requiring two divalent cations such as Mn^2+^, Co^2+^, or Zn^2+^ for maximal activity (Kitchener & Grunden, 2012). Prolidase is a member of the pita-bread enzyme family (Lowther & Matthews, 2002), which contains dinuclear metal clusters coordinated by identical sets of amino acid residues (two Glu, two Asp, and one His). A previous examination of the crystal structure of *Ec*PepQ has demonstrated that the enzyme features five conservative metal binding residues (Asp246, Asp257, His339, Glu384, and Glu423) to chelate two metal ions (Weaver et al., 2014). Consistently, *Ec*PepQ required Mn^2+^ ion for maximum activity (Fig. 4). Stimulation by Mn^2+^ ion has also been observed in other prolidases from both prokaryotic and eukaryotic organisms (Jalving et al., 2002; Lupi et al., 2006; Vyas et al., 2010; Theriot et al., 2010; Huang & Tanaka, 2015).

Microbial enzymes have gained a lot of interest for their widespread uses in a vast array of industries (Singh et al., 2016). However, the stability of enzymes is always a key challenge on the implementation of the biocatalysts in industrial processes, which are designed to operate under extreme temperatures (Silva et al., 2018). Thus, it is imperative to understand as many as possible about how an enzyme loses stability and to what extent we can more precisely control its ideal temperature for catalysis. Interestingly enough, the recombinant prolidase from *E. coli* NovaBlue, a mesophilic bacterium, had a temperature optimum of 60 °C and a well-defined unfolding transition of 64.2 °C (Figs. 3 and 5). This would enable us to perform *Ec*PepQ-mediated catalysis at high temperatures. Advantages for a biocatalytic process operated at high temperatures have been reported in the literature (Iyer & Ananthanarayan, 2008).

Guanidine hydrochloride is a chemical denaturant that has been widely used to denature proteins and characterize the conformation, stability, and folding/unfolding pathway and mechanism of proteins (Povarova, Kuznetsova & Turoverov, 2010; England & Haran, 2011). Therefore, we also studied the effect of GdnHCl on the enzymatic activity and unfolding of *Ec*PepQ. The activity assay and inactivation kinetics suggest the GdnHCl-induced inactivation of *Ec*PepQ is a monophasic and concentration-dependent process (Fig. 6). The increase in AEW observed up to 1.2M GdnHCl may be caused by tertiary structural rearrangement involving aromatic residues or because of the increased mobility of the local environment of the aromatic residues. Normally, exposed aromatic residues in the unfolded proteins show emission maxima between 348 and 356 nm (Lackowicz, 2006). Treatment of *Ec*PepQ with higher concentrations of GdnHCl has resulted in exposure of buried residues of the native enzyme to the solvent as a red shift in AEW (from 345 to 356 nm).

Effect of the tested organic co-solvents on the prolidase activity of *Ec*PepQ was relatively complex and difficult to precisely elucidate at this stage. However, several key factors, including alterations in conformation and flexibility of enzymes, (de)solvation of active sites, energetics of substrate desolvation, steric hindrance that restricts the accessibility of substrate, and competitive inhibition by co-solvent molecules, are probably responsible for the inactivation of enzymes in the presence of water-miscible organic co-solvents (Kim, Clark & Dordick, 2000; Klibanov, 2001; Graber et al., 2007). In spite of the fact that most of the employed organic co-solvents are strong denaturants, *Ec*PepQ was still clearly active in several water/organic co-solvent mixtures at high concentrations (Fig. 7). This intrinsic capability has also been discovered in a variety of enzymes, including glyceraldehyde-3-phosphate dehydrogenase (Wiggers et al., 2007), carboxylesterase (Mandrich et al., 2012), lipase (Park et al., 2005), NADH oxidase (Toth et al., 2010), and hydrogenase (Serebryakova, Zorin & Karyakin, 2009).

As shown in Fig. 8, organic co-solvent concentrations that caused a dramatic loss of the prolidase activity of *Ec*PepQ was generally detrimental to the molecular structure of the enzyme. The denaturation of proteins by organic co-solvents can be referred to the disruption of the hydration shell around the biomacromolecules or the distortion of the hydrophobic interactions that help to keep the correct folding of proteins (Serdakowski & Dordick, 2008). Thus, a conformational change in the molecular structure may be the primary reason for the inactivation of *Ec*PepQ under higher concentrations of acetonitrile, methanol, and ethanol. This is closely linked with other previous investigations that show a pretty strong relationship between the loss of catalytic activity and the extent of structural integrity of enzymes in water/organic co-solvents mixtures (Tsuzuki, Ue & Nagao, 2003; Secundo et al., 2011). It is also noteworthy that the protein structure of *Ec*PepQ *did not alter profoundly* in the presence of 50% glycerol, 60% ethylene glycol, 15% THF, and 30% isopropanol (Fig. 8). Several previous reports have made great strides toward causes of activity loss in organic co-solvents (Klibanov, 1997; Kim, Clark & Dordick, 2000; Klibanov, 2001; Graber et al., 2007). Based on these elucidations, other factors instead of structural changes can possibly contribute to the great loss of *Ec*PepQ activity in glycerol, ethylene glycol, THF, and isopropanol.

## Conclusion

In summary, a His_6_-tagged prolidase from *E. coli* NovaBlue was overexpressed, purified to an electrophoretic purity of ∼96%, and characterized at a molecular level. Similar to most organophosphate-degrading prolidases, the dimeric enzyme was very active in the presence of Mn^2+^ ion. However, it should be emphasized that a clear change in the protein conformation of *Ec*PepQ was observed upon heat and GdnHCl treatments. These insights into structure–function relationships can guide the protein engineering of *Ec*PepQ to enable the production of more stable variants. Furthermore, a rather unexpected outcome is the fairly high tolerance of *Ec*PepQ toward the tested organic co-solvents. This information is definitely valuable for potential future applications of *Ec*PepQ, particularly for performing its biocatalysis in water/organic co-solvent systems.

## Supplemental Information

10.7717/peerj.5863/supp-1 Supplemental Information 1 Raw data for the specific activity of E. coli M15 (pQE-EcPepQ) under a specific condition

10.7717/peerj.5863/supp-2 Supplemental Information 2 Analysis of the cell-free extracts of E .coli (pQE-EcPepQ) by SDS-PAGE

10.7717/peerj.5863/supp-3 Supplemental Information 3 Analysis of the soluble proteins of E. coli M15 (pQE-EcPepQ) by SDS-PAGE

10.7717/peerj.5863/supp-4 Supplemental Information 4 Raw data for FPLS analysis

10.7717/peerj.5863/supp-5 Supplemental Information 5 Raw data for analysis of the native enzyme by non-denaturing PAGE

10.7717/peerj.5863/supp-6 Supplemental Information 6 Raw data for effect of temperature on activity and stability of EcPepQ

10.7717/peerj.5863/supp-7 Supplemental Information 7 Raw data for effect of pH on activity and stability of EcPepQ

10.7717/peerj.5863/supp-8 Supplemental Information 8 Raw data for effect of divalent metal ions on the catalytic activity of EcPepQ

10.7717/peerj.5863/supp-9 Supplemental Information 9 Effect of different concentrations of Mn2+ ion on the catalytic activity of EcPepQ

10.7717/peerj.5863/supp-10 Supplemental Information 10 Raw data for transition and cooling curves of the enzyme

10.7717/peerj.5863/supp-11 Supplemental Information 11 Raw data for far-UV CD and intrinsic tryptophan fluorescence spectra of EcPepQ

10.7717/peerj.5863/supp-12 Supplemental Information 12 Raw data for concentration effect of GdnHCl on the catalytic activity of EcPepQ

10.7717/peerj.5863/supp-13 Supplemental Information 13 Raw data for concentration effect of GdnHCl on the corresponding changes in the tertiary structure as monitored by AEW value

10.7717/peerj.5863/supp-14 Supplemental Information 14 Raw data for effect of different water-miscible organic co-solvents on the enzynatic activity of EcPepQ

10.7717/peerj.5863/supp-15 Supplemental Information 15 Raw data for far-UV CD and intrinsic tryptophan fluorescence spectra of EcPepQ in the presence of water-miscible organic co-solvents

## Additional Information and Declarations

### Data Availability

The following information was supplied regarding data availability: The raw data are provided in the Supplemental files.

## References

[ref-1] Arakawa T, Kita Y, Timasheff SN (2007). Protein precipitation and denaturation by dimethyl sulfoxide. Biophysical Chemistry.

[ref-2] Batista ANL, Batista JM, Bolzani VS, Furlan M (2013). Selective DMSO-induced conformational changes in proteins from Raman optical activity. Physical Chemistry Chemical Physics.

[ref-3] Browne P, O’Cuinn G (1983). The purification and characterization of a proline dipeptidase from guinea pig brain. Journal of Biological Chemistry.

[ref-4] Chandrasekaran L, Belinskaya T, Saxena A (2013). *In vitro* characterization of organophosphorus compound hydrolysis by native and recombinant human prolidase. Toxicology in Vitro.

[ref-5] Cheng TC, Rastogi VK, Defrank JJ, Sawiris GP (1998). G-type nerve agent decontamination by *Alteromonas* prolidase. Annals of the New York Academy of Sciences.

[ref-6] Endo F, Hata A, Indo Y, Motohara K, Matsuda I (1987). Immunochemical analysis of prolidase deficiency and molecular cloning of cDNA for prolidase of human liver. Journal of Inherited Metabolic Disease.

[ref-7] DeFrank JJ, Cheng TC (1991). Purification and properties of an organophosphorus acid anhydrase from a halophilic bacterial isolate. Journal of Bacteriology.

[ref-8] DiTargiani RC, Chandrasekaran L, Belinskaya T, Saxena A (2010). In search of a catalytic bioscavenger for the prophylaxis of nerve agent toxicity. Chemico-Biological Interactions.

[ref-9] England JL, Haran G (2011). Role of solvation effects in protein denaturation: from thermodynamics to single molecules and back. Annual Review of Physical Chemistry.

[ref-10] Fernandez-Espla MD, Martin-Hernandez MC, Fox PF (1997). Purification and characterization of a prolidase from *Lactobacillus casei* subsp. casei IFPL 731. Applied and Environmental Microbiology.

[ref-11] Ganesan K, Raza SK, Vijayaraghavan R (2010). Chemical warfare agents. Journal of Pharmacy & Bioallied Sciences.

[ref-12] Ghosh M, Grunden AM, Dunn DM, Weiss R, Adams MW (1998). Characterization of native and recombinant forms of an unusual cobalt-dependent proline dipeptidase (prolidase) from the hyperthermophilic archaeon *Pyrococcus furiosus*. Journal of Bacteriology.

[ref-13] Graber M, Irague R, Rosenfeld E, Lamare S, Franson L, Hult K (2007). Solvent as a competitive inhibitor for *Candida antarctica* lipase B. Biochimica et Biophysica Acta.

[ref-14] Hasimoto K, Panchenko AR (2010). Mechanisms of protein oligomerization, the critical role of insertions and deletions. Proceedings of the National Academy of Sciences of the United States of America.

[ref-15] Hu CA, Phang JM, Valle D (2008). Proline metabolism in health and disease: preface. Amino Acids.

[ref-16] Huang P, Dong A, Caughey WS (1995). Effects of dimethyl sulfoxide, glycerol, and ethylene glycol on secondary structures of cytochrome *c* and lysozyme as observed by infrared spectroscopy. Journal of Pharmaceutical Sciences.

[ref-17] Huang Y, Tanaka T (2015). Characterization of two putative prolidases (PrpR1 and PepR2) from *Lactobacillus plantarum* WCFS1: occurrence of two isozymes with structural similarity and different properties. Biochimica et Biophysica Acta.

[ref-18] Iyer PV, Ananthanarayan L (2008). Enzyme stability and stabilization—aqueous and non-aqueous environment. Process Biochemistry.

[ref-19] Jalving R, Bron P, Kester HC, Visser J, Schaap PJ (2002). Cloning of a prolidase gene from *Aspergillus nidulans* and characterization of its product. Molecular Genetics and Genomics.

[ref-20] Jeyakanthan J, Takada K, Sawano M, Ogasahara K, Mizutani H, Kunishima N, Yokoyama S, Yutani K (2009). Crystal structural and functional analysis of the putative dipeptidase from *Pyrococcus horikoshii* OT3. Journal of Biophysics.

[ref-21] Karpouzas DG, Singh BK (2006). Microbial degradation of organophosphorus xenobiotics: metabolic pathways and molecular basis. Advances in Microbial Physiology.

[ref-22] Kaur J, Kumar A, Kaur J (2018). Strategies for optimization of heterologous protein expression in *E. coli*: roadblocks and reinforcements. International Journal of Biological Macromolecules.

[ref-23] Kgosisejo O, Chen JA, Grochulski P, Tanaka T (2017). Crystallographic structure of recombinant *Lactococcus lactis* prolidase to support proposed structure-function relationships. Biochimica et Biophysica Acta.

[ref-24] Kim J, Clark DS, Dordick JS (2000). Intrinsic effects of solvent polarity on enzymic activation energies. Biotechnology and Bioengineering.

[ref-25] Kitchener RL, Grunden AM (2012). Prolidase function in proline metabolism and its medical and biotechnological applications. Journal of Applied Microbiology.

[ref-26] Klibanov AM (1997). Why are enzymes less active in organic solvents than in water?. Trends in Biotechnology.

[ref-27] Klibanov AM (2001). Improving enzymes by using them in organic solvents. Nature.

[ref-28] Kumar SV, Fareedullah M, Sudhakar Y, Venkateswarlu B, Kumar EA (2010). Current review on organophosphorus poisoning. Archives of Applied Science Research.

[ref-29] Lackowicz JR (2006). Principles of Fluorescence Spectroscopy.

[ref-30] Liu S (2015). A review on protein oligomerization process. International Journal of Precision and Engineering Manufacturing.

[ref-31] Lobley A, Whitmore L, Wallace BA (2002). DICHROWEB: an interactive website for the analysis of protein secondary structure from circular dichroism spectra. Bioinformatics.

[ref-32] Lowther WT, Matthews BW (2002). Metalloaminopeptidases: common functional themes in disparate structural surroundings. Chemical Reviews.

[ref-33] Lupi A, Della Torre S, Campari E, Tenni R, Cetta G, Rossi A, Forlino A (2006). Human recombinant prolidase from eukaryotic and prokaryotic sources: expression, purification, characterization and long-term stability studies. FEBS Journal.

[ref-34] Maher MJ, Ghosh M, Grunden AM, Menon AL, Adams MW, Freeman HC, Guss JM (2004). Structure of the prolidase from *Pyrococcus furiosus*. Biochemistry.

[ref-35] Mandrich L, De Santi C, De Pascale D, Manco G (2012). Effect of low organic solvents concentration on the stability and catalytic activity of HSL-like carboxylesterases: analysis from psychrophiles to (hyper)thermophiles. Journal of Molecular Catalysis B: Enzymatic.

[ref-36] Namiduru ES (2016). Prolidase. Bratislava Medical Journal.

[ref-37] Pace CN (1990). Measuring and increasing protein stability. Trends in Biotechnology.

[ref-38] Marianayagam NJ, Sunde M, Matthews JM (2004). The power of two: protein dimerization in biology. Trends in Biochemical Sciences.

[ref-39] Park MS, Hill CM, Li Y, Hardy RK, Khanna H, Khang YH, Raushel FM (2004). Catalytic properties of the PepQ prolidase from *Escherichia coli*. Archives of Biochemistry and Biophysics.

[ref-40] Park H, Lee KS, Chi YM, Jeong SW (2005). Effects of methanol on the catalytic properties of porcine pancreatic lipase. Journal of Microbiology and Biotechnology.

[ref-41] Povarova OI, Kuznetsova IM, Turoverov KK (2010). Differences in the pathways of proteins unfolding induced by urea and guanidine hydrochloride: molten globule state and aggregates. PLOS ONE.

[ref-42] Rosano GL, Ceccarelli EA (2014). Recombinant protein expression in *Escherichia coli*: advances and challenges. Frontiers in Microbiology.

[ref-43] Royer CA (2006). Probing protein folding and conformational transitions with fluorescence. Chemical Reviews.

[ref-44] Royer CA, Mann CJ, Matthews CR (1995). Resolution of the fluorescence equilibrium unfolding profile of trp aporepressor using single tryptophan mutants. Protein Science.

[ref-45] Schenk G, Mateen I, Ng TK, Pedroso MM, Mitić N, Jafelicci M, Marques RFC, Gahan LR, Ollis DL (2016). Organophosphate-degrading metallohydrolases: structure and function of potent catalysts for applications in bioremediation. Coordination Chemistry Reviews.

[ref-46] Secundo F, Fiala S, Fraaije MW, De Gonzalo G, Meli M, Zambianchi F, Ottolina G (2011). Effects of water miscible organic solvents on the activity and conformation of the Baeyer-Villiger monooxygenases from *Thermobifida fusca* and *Acinetobacter calcoaceticus*: a comparative study. Biotechnology and Bioengineering.

[ref-47] Serdakowski AL, Dordick JS (2008). Enzyme activation for organic solvents made easy. Trends Biotechnology.

[ref-48] Serebryakova LT, Zorin NA, Karyakin AA (2009). Improvement of hydrogenase enzyme activity by water-miscible organic solvents. Enzyme and Microbial Technology.

[ref-49] Silva C, Martins M, Jiang S, Fu J, Cavaco-Paulo A (2018). Practical insights on enzyme stabilization. Critical Reviews in Biotechnology.

[ref-50] Singh BK (2009). Organophosphorus-degrading bacteria: ecology and industrial applications. Nature Reviews Microbiology.

[ref-63] Singh R, Kumar M, Mittal A, Methta PK (2016). Microbial enzymes: industrial progress in 21st century. 3 Biotech.

[ref-51] Sjöström H, Noŕen O, Josefsson L (1973). Purification and specificity of pig intestinal prolidase. Biochimica et Biophysica Acta.

[ref-52] Suga K, Kabashima T, Ito K, Tsuru D, Okamura H, Kataoka J, Yoshimoto T (1995). Prolidase from *Xanthomonas maltophilia*: purification and characterization of the enzyme. Bioscience, Biotechnology and Biochemistry.

[ref-53] Timasheff SN (2002). Protein-solvent preferential interactions, protein hydration, and the modulation of biochemical reactions by solvent components. Proceedings of the National Academy of Sciences of the United States of America.

[ref-54] Theriot CM, Du X, Tove SR, Grunden AM (2010). Improving the catalytic activity of hyperthermophilic *Pyrococcus* prolidases for detoxification of organophosphorus nerve agents over a broad range of temperatures. Applied Microbiology and Biotechnology.

[ref-55] Theriot CM, Semcer RL, Shah SS, Grunden AM (2011). Improving the catalytic activity of hyperthermophilic *Pyrococcus horikoshii* prolidase for detoxification of organophosphorus nerve agents over a broad range of temperatures. Archaea.

[ref-56] Toth K, Sedlak E, Musatov A, Zoldak G (2010). Activity of NADH oxidase from *Thermus thermophilus* in water/alcohol binary mixtures is limited by the stability of quaternary structure. Journal of Molecular Catalysis B: Enzymatic.

[ref-57] Tsuzuki W, Ue A, Nagao A (2003). Polar organic solvent added to an aqueous solution changes hydrolytic property of lipase. Bioscience, Biotechnology and Biochemistry.

[ref-58] Vyas NK, Nickitenko A, Rastogi VK, Shah SS, Quiocho FA (2010). Structural insights into the dual activities of the nerve agent degrading agent organophosphate anhydrolase/prolidase. Biochemistry.

[ref-59] Weaver J, Watts T, Li P, Rye HS (2014). Structural basis of substrate selectivity of *E. coli* prolidase. PLOS ONE.

[ref-60] Wiggers HJ, Cheleski J, Zottis A, Oliva G, Andripulo AD, Montanari CA (2007). Effects of organic solvents on the enzyme activity of *Trypanosoma cruzi* glyceraldehyde-3-phosphate dehydrogenase in calorimetric assays. Analytical Biochemistry.

[ref-61] Yang SI, Tanaka T (2008). Characterization of recombinant prolidase from *Lactococcus lactis*: changes in substrate specificity by metal cations and allosteric behavior of the prolidase. FEBS Journal.

[ref-62] Yuh H, Lee S, Kim S, Yu J, Lee N, Lee J, Kim ND, Yu C, Rho J (2017). Improved hydrolysis of organophosphorus compounds by engineered human prolidases. Protein & Peptide Letters.

