# Supplementary figures and images for: High-level expression and molecular characterization of a recombinant prolidase from Escherichia coli NovaBlue
Source: PeerJ. 2018 Oct 31;6:e5863. doi: 10.7717/peerj.5863 (PMC6215446; doi:10.7717/peerj.5863)

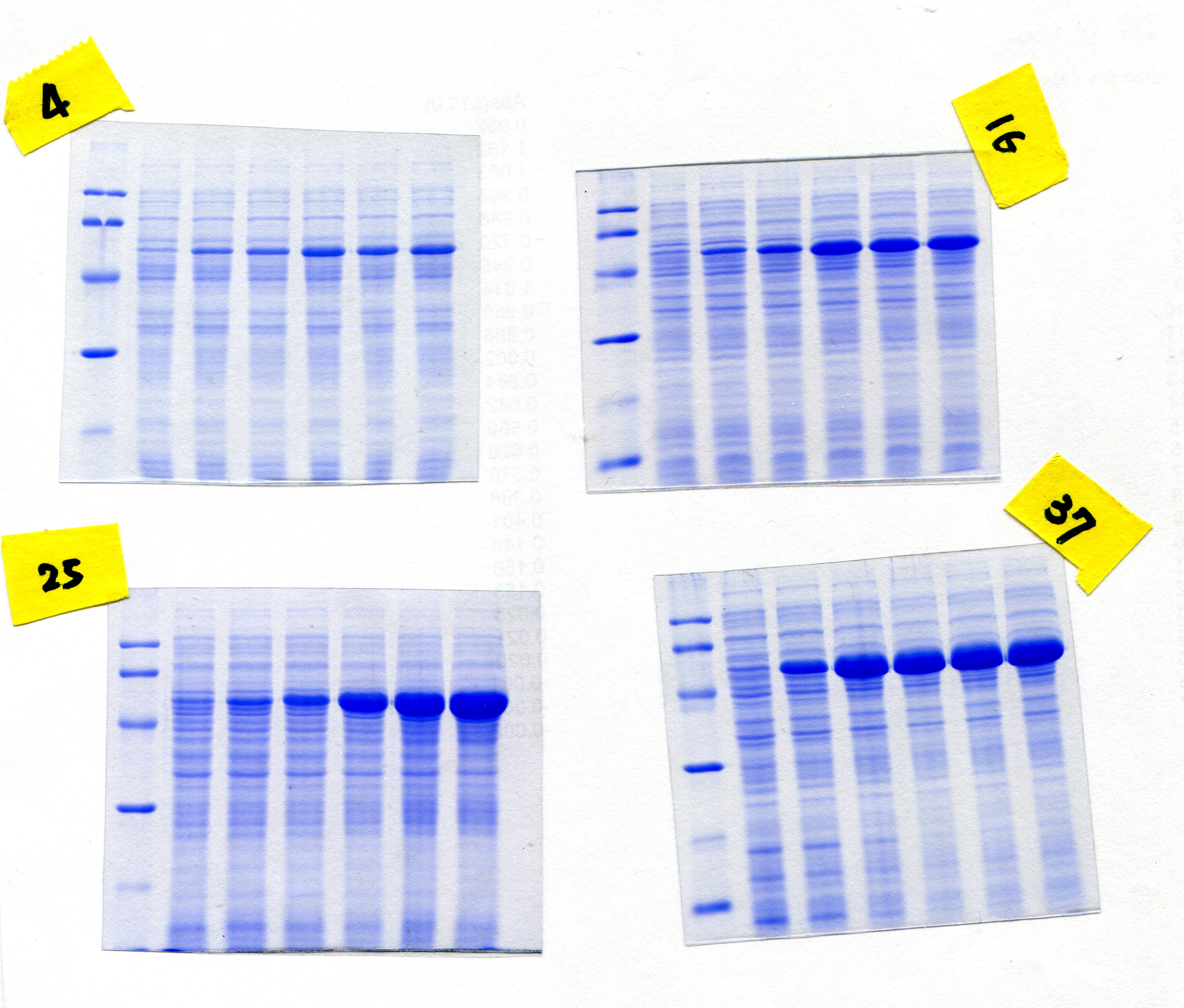

Supplement: Supplemental Information 2 [file peerj-06-5863-s002.jpg]

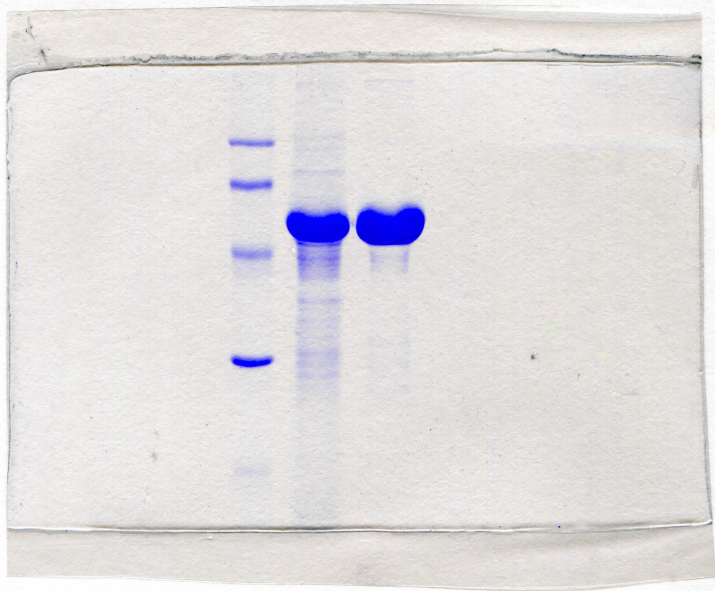

Supplement: Supplemental Information 3 [file peerj-06-5863-s003.pdf]

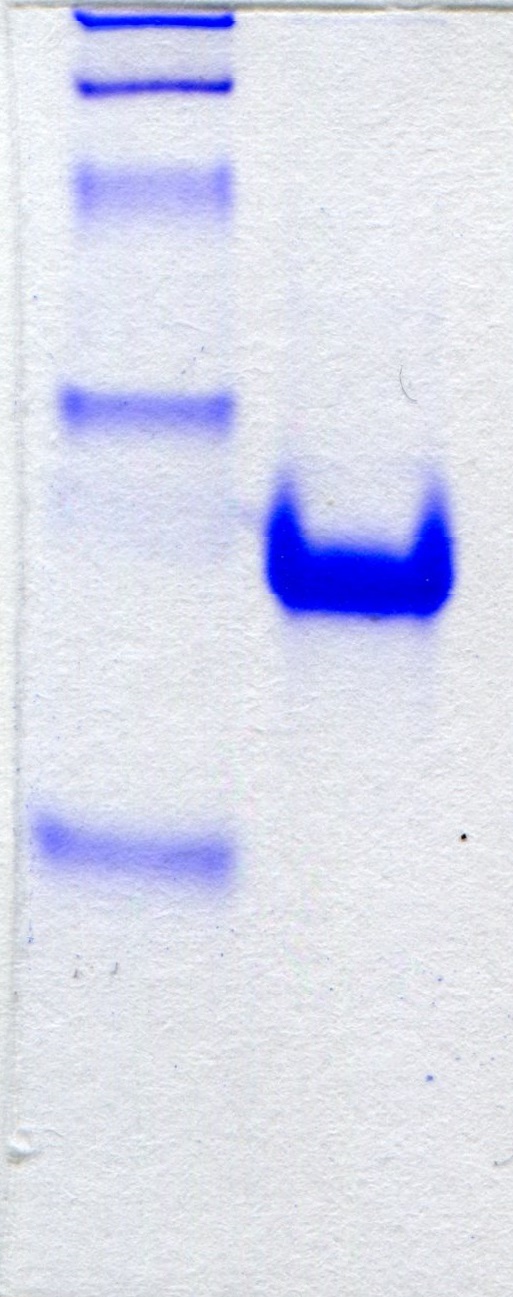

Supplement: Supplemental Information 5 [file peerj-06-5863-s005.jpg]
